# Protective effects of thalidomide on pulmonary injuries in a rat model of paraquat intoxication

**DOI:** 10.1186/s12950-015-0093-0

**Published:** 2015-07-28

**Authors:** Dan Li, Xiao-Wei Zhang, Xing-Quan Jiang, Yong-Jie Yin, Zhe Fan, Cheng-Bo Sun, Xing-Hai Chen, Yan-Hui Li, Ke-Xiang Liu

**Affiliations:** Department of Emergency Medicine, The Second Hospital of Jilin University, No. 218 Ziqiang Street, Changchun, 130041 Nanguan District People’s Republic of China; Department of Cardiovascular Surgery, The Second Hospital of Jilin University, No. 218 Ziqiang Street, Changchun, 130041 Nanguan District People’s Republic of China; Department of Clinical Laboratory, Children’s Hospital of Changchun City, No. 69 Beian Road, Changchun, 130061 Chaoyang District People’s Republic of China; Department of Technology, People’s Procuratorate of Jilin Province, No. 1333 Jingyang Road, Changchun, 130062 Lvyuan District People’s Republic of China; Department of Experimental Pharmacology and Toxicology, School of Pharmacy, Jilin University, No. 1163 Xinmin Avenue, Changchun, 130021 Chaoyang District People’s Republic of China

**Keywords:** Paraquat, Lung injury, Thalidomide, Protective effects

## Abstract

**Background:**

This study was designed to evaluate the protective effects of thalidomide on paraquat (PQ)-induced lung injuries in a rat model and to explore the underlying mechanisms.

**Methods:**

Rats were exposed to 50 mg/kg PQ by oral gavage, and treated with thalidomide through oral administration at 60 mg/kg once a day, 6 days/week for 2 weeks. Serum levels of IL-6, TNF-alpha, TGFbeta1 and COL1A1 were detected at different time points after paraquat exposure. At the end of the study, lung tissues were collected for pathological inspection as well as analyses of water content and expression levels of IL-6, TNF-alpha, TGFbeta1 and COL1A1 mRNA.

**Results:**

The results showed that thalidomide treatment could significantly alleviate PQ-induced pathological changes in lung tissue and severity of lung edema. Thalidomide treatment after PQ exposure resulted in significantly reduced serum levels of IL-6, TNF-alpha, TGF-beta1 and COL1A1, as compared to PQ group. PCR analysis demonstrated that expression levels of IL-6, TNF-alpha, TGF-beta1 and COL1A1 in lung tissue were significantly increased after PQ exposure but reduced by thalidomide, which were confirmed by immunohistochemistry staining.

**Conclusions:**

Our results indicated that inflammatory factors played important roles in PQ-induced lung injuries and thalidomide could protect rats from PQ-induced lung injuries by inhibiting the upregulation of inflammatory factors.

## Background

Paraquat (1,1′-dimethyl-4,4′-bipyridilium dichloride, PQ) is a widely used nonselective herbicide around the world and the incidence rate of PQ intoxication has been reported to be increasing. The primary damages caused by PQ occur in lung tissue due to the accumulation of PQ. PQ exposure results in acute damage and destruction of alveolar epithelial cells, pulmonary edema, and infiltration of inflammatory cells within a few days. The acute injury phase is then followed by a final pulmonary fibrotic phase that lasts for several weeks featured by infiltration of myofibroblasts into the alveolar spaces and septa, and differentiation in fibroblasts with the production of collagen [1]. Pulmonary fibrosis is a major hallmark and a leading cause of death in PQ intoxication. The high mortality rate of PQ-intoxicated patients imposes a challenge to clinical practitioners due to the lack of an antidote or effective treatment to prevent pulmonary fibrosis [2].

A number of studies have suggested that the PQ-induced oxidative stress is the primary mechanism for initiating lung damage by PQ. PQ intoxication generates numerous oxygen free radical species by cyclic oxido-reduction of PQ, which cause disruptions of alveolar epithelial cells and Clara cells, infiltration of inflammatory cells into the interstitial and alveolar spaces, upregulation of several genes involved in inflammatory response, proliferation of fibroblasts, and deposition of excessive collagen [1, 3]. Several inflammatory cytokines, particularly TNF-α, IL-6, IL-1β, and TGF-β1 are found to play key roles in the pathogenesis of PQ-induced lung injury and fibrosis [4, 5]. Although there is no effective therapy for PQ poisoning, anti-inflammatory drugs including corticosteroids and immunosuppressive drugs have been used in the clinical treatment for PQ intoxication [6, 7].

Thalidomide (Thal), originally developed in the 1950s as a tranquilizer, was discontinued for its potent teratogenic effects in the 1960s. It has recently being shown to have various pharmacological properties, including immunomodulation [8], anti-inflammation [9–11], and anti-angiogenesis [12]. Thalidomide and its analogs have been used in the treatment of a variety of disorders including erythema nodosum leprosum, multiple myeloma, rheumatoid arthritis, Crohn’s disease, prostate cancer and lupus erythematosus [13]. In recent experimental studies, thalidomide has been demonstrated to have anti-fibrotic effects by suppressing the expression of IL-6, TGF-β, and angiogenesis-related growth factors that play a crucial role in the proliferation and differentiation of lung fibroblasts [14].

Based on the fact that inflammatory mediators contribute greatly to PQ-induced acute lung injury and subsequent fibrosis, we hypothesize that thalidomide may be potentially used to relieve PQ-induced pulmonary inflammation and fibrosis. In the present study, we explored whether Thal has protective effects on PQ-induced lung injury and fibrosis using PQ intoxication rat model. Moreover, we also investigated the mechanisms underlying the therapeutic effect of thalidomide on pulmonary inflammation and fibrosis.

## Methods

### Reagents

Paraquat was provided by Shandong Yinuo Company (Shandong, China); Thalidomide was purchased from Changzhou Pharmaceutical Company (Changzhou, China); Polyclonal antibodies against TNF-α, IL-6, TGFβ1 and collagen-1 were purchased from Boster Biological Technology (Wuhan, China); Horseradish peroxidase (HRP)-conjugated goat anti-rabbit secondary antibody and streptavidin-peroxidase immunohistochemistry kit were provided by Beijing Zhongshan-Golden Bridge Biological Technology (Beijing, China); Trizol and SuperScript II Reverse Transcriptase were obtained from Invitrogen (Carlsbad, USA); Taq DNA polymerase and DNA size marker were purchased from Beijing Dingguo Changsheng Biotechnology (Beijing, China).

### Animal model and experimental procedures

All animal studies were performed in accordance with the guidelines of the Jilin University and the animals received human care in compliance with the Principles of Laboratory Animal Care. Healthy female Wistar rats weighing 250 ~ 300 g purchased from Animal Research Center of Jilin University were hosted in a pathogen-free animal facility under a standard 12-h light/12-h dark cycle. Rats were fed standard rodent chow and water ad libitum.

Rats were randomly assigned to three groups: (1) normal control group, five rats with oral administration of 1 ml of 0.9 % NaCl solution only; (2) PQ group, 14 rats with one time oral administration of paraquat (50 mg/kg) in 1 ml of saline; and (3) PQ+ Thal group, ten rats with one time oral administration of paraquat (50 mg/kg) in 1 ml of saline followed by oral gavage of thalidomide at 60 mg/kg once a day, 6 days/week for 2 weeks. Blood samples were collected through retro-orbital bleeding at days 1, 2, 3, 5, 7, 10 and 15 after paraquat exposure for detection of IL-6, TNF-α, TGFβ1 and COL1A1 levels. Rats were euthanized at day 15 and lung tissues were collected for analyses of water content and expression levels of IL-6, TNF-α and COL1A1 mRNA in lung as well as pathological inspection.

### Detection of serum IL-6, TNF-α, TGFβ1 and COL1A1 levels

The concentrations of IL-6, TNF-α, TGFβ1 and COL1A1 were determined using ELISA. Briefly, 96-well, flat bottom plates were coated with serum samples overnight at 4 °C. The plates were washed and blocked with PBS with 10 % fetal bovine serum for 1.5 h. After the plates were washed, primary detection antibodies specific to IL-6, TNF-α, TGFβ1 and COL1A1 were added to the respective wells for 1.5 h. The plates were washed and horseradish peroxidase (HRP)-conjugated secondary antibody was added to the plates for 1.5 h. Following a series of stringent washing, the substrate reagent was added into each well and absorbance was read at 490 nm using the plate reader. The detection limits of ELISA methods for IL-6, TNF-α, TGFβ1 and COL1A1 were all below 50 pg/ml.

### Histological examinations of the lungs

The lung tissues from each treatment group were excised, fixed in 10 % buffered formalin and then embedded in paraffin. Sliced lung tissue sections (2 μm in thickness) were stained with Hematoxylin-and-Eosin (H&E) and pathological changes were examined by experienced pathologists. Masson’s trichrome staining was used to assess collagen deposition in lung extracellular matrix.

### Detection of mRNA expression levels of IL-6, TNF-α, TGFβ1 and COL1A1 in the lungs by Reverse Transcription-Polymerase Chain Reaction (RT-PCR)

Total RNA from rat lung tissue was extracted using Trizol (Invitrogen, California, USA) by following manufacturer’s instruction. cDNA was synthesized from 2 μg of total RNA by using SuperScript II reverse transcription kit (Invitrogen, California, USA) for each sample. Conventional PCR was used to detect expression levels of IL-6, TNF-α, TGFβ1 and COL1A1 with GAPDH used as endogenous control. The specific primer sets (Table 1) were designed based on sequences deposited in the NCBI GenBank database. The thermal cycling conditions included 1 cycle at 94 °C for 2 min and 35 cycles at 94 °C for 1 min, 50 °C for 1 min and 72 °C for 1 min followed by 1 cycle at 72 °C for 10 min. PCR products were resolved by electrophoresis on 1.5 % agarose gel and images were collected using gel imaging system.

**Table 1:** Primers used for the PCR detection of specific gene expression

Gene	Forward	Reverse	PCR product size
*TGF-β1*	5′-GACTCCTGCTGCTTT CTCC-3′	5′-GCGGTCCACCATTA GCAC-3′	531 bp
*IL-6*	5′-CCACGGCCTTCCCTA CTTC-3′	5′-TTGGTCCTTAGCCAC TCCT-3′	499 bp
*COL1A1*	5′-GTCCTATGGCTATGATG AGAA ATC-3′	5′-CACCATCCAAACCA CTGAAAC-3′	335 bp
*TNF-a*	5′-GTCCCCAAAGGGATGA GAAG T-3′	5′-TGAGATAGCCAAATCG GCT GAC-3′	323 bp
*GAPDH*	5′-ACCACAGTCCATGCCAT CAC-3′	5′-TCCACCACCCTGTTGCT GTA-3′	452 bp

### Immunohistochemistry detection of IL-6, TNF-α, TGFβ1 and COL1A1 expression in the lungs

Immunohistochemical staining (IHC) was performed using streptavidin-peroxidase immunohistochemistry kit by following the instructions. Rabbit polyclonal antibodies against TNF-α, IL-6, TGFβ1 and COL1A1 were used as primary antibodies, and equal amount of purified rabbit IgG was used as negative control. The signal was developed using DAB substrate.

### Statistical analysis

All data were presented as the mean ± standard differences (S.D.). Data were analyzed by one-way analysis of variance (ANOVA) with Bonferroni post-tests for comparison between groups. In all cases, the difference was considered statistically significant as *P* < 0.05.

## Results

### Effects of thalidomide on PQ-induced morbidity and mortality

The rats in PQ group presented with tachypnea, labored breathing, and anorexia within 2 to 3 days post PQ exposure. Two out of 14 rats died on day 2 and one died on day 3 after PQ administration. Severe interstitial edema, alveolar hemorrhage, and inflammation in all alveolar interstitial tissues and lumens were observed at necropsy (data not shown). In contrast, no apparent signs of respiratory distress were observed in rats in PQ + Thal group after PQ exposure and no animal died in this group.

### Effect of thalidomide on lung water content

Lung water content was calculated by the formula of (lung wet weight-lung dry weight)/lung dry weight. Lung water content was higher in PQ group at day 15 post PQ exposure compared to control group and was significantly reduced in the PQ + Thal group compared to PQ group (*p* < 0.05) (Table 2). This indicated that lung edema remained for a long period of time following PQ intoxication, and treatment with thalidomide could effectively relieve PQ-induced lung edema.

**Table 2:** Water content of lung tissues at the end of experiment

Group	Lung water content
Normal control	4.94 ± 0.24
PQ	5.21 ± 0.72
PQ + Thal	4.63 ± 0.54*

Data are presented as **(M**ean ± SD)

*P < 0.05, compared to PQ

### Thalidomide alleviated the histopathological changes of lung caused by PQ

**Fig. 1:**
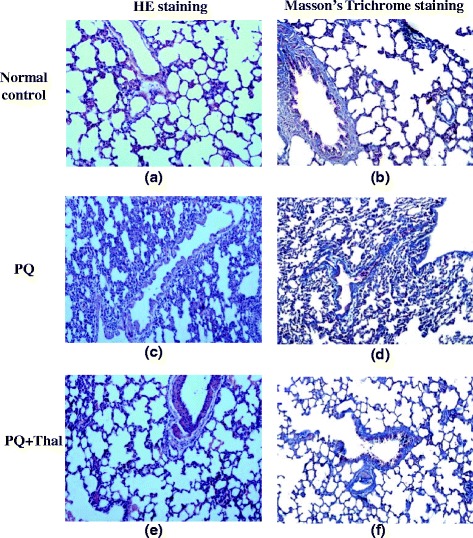
Thalidomide alleviated PQ induced histopathological changes in lung tissues in rats. Lung tissues from Control (**a**, **b**), PQ (**c**, **d**) and PQ + Thal (**e**, **f**) groups were subjected to pathological examination on day 15 after PQ administration by hematoxylin and eosin staining (×100) (**a**, **c**, **e**) and Masson’s trichrome staining (×100) (**b**, **d**, **f**)

PQ-induced lung structural changes and alleviative effects of thalidomide on PQ-induced damages are depicted in Fig. 1. Histological changes were assessed with H&E staining and lung fibrosis was identified by using Masson’s Trichrome stain for collagen. Animals from control group presented a normal pulmonary structure at light microscopy, without evidences of alveolar collapse, cellular infiltrations, or collagen accumulation. Paraquat intoxicated rat lung on day 15 showed diffuse alveolar damages with thickening of alveolar walls and widespread inflammation in alveolar spaces and septa. There were significant peribronchial and perialveolar deposition of collagen observed in Masson’s trichrome staining. In comparison with the PQ group, the occurrence of the above, referred alterations were drastically attenuated in the PQ + Thal groups, particularly inflammation, hemorrhage, and the amount of accumulation of collagenous fiber.

### Effects of thalidomide on serum levels of IL-6, TNF-α, TGF-β1 and COL1A1 induced by PQ

**Fig. 2:**
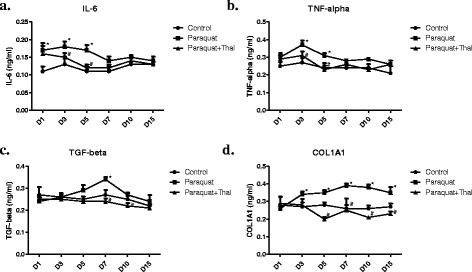
Effects of thalidomide on serum levels of IL-6, TNF-α, TGF-β1 and COL1A1 induced by PQ. Blood samples from rats from each treatment group were collected through retro-orbital bleeding at days 1, 2, 3, 5, 7, 10 and 15 after paraquat exposure and serum levels of IL-6 (**a**), TNF-α (**b**), TGF-β1 (**c**) and COL1A1 (**d**) were measured by ELISA. The values represent mean ± SD from each group (● Control, ■ PQ group and ▲ PQ + Thal group). * p <0.05 compared to Control, # p <0.05 compared to Paraquat

Serum levels of IL-6, TNF-α, TGF-β1 and COL1A1 were measured by ELISA on day 1, 3, 5, 7, 10 and 15 after PQ exposure. In PQ group, serum IL-6 levels significantly elevated on day 1 compared to control group, peaked on day 3, and gradually reduced thereafter. Thalidomide treatment resulted in accelerated reduction of PQ-induced elevation of IL-6 levels (Fig. 2a).

Serum TNF-α levels increased in PQ group on day 1 post PQ exposure, peaked on day 3, and remained higher than control group on day 5. Rats treated with thalidomide following PQ exposure had significantly reduced serum TNF-α levels on day 3 and 5 compared to PQ group (Fig. 2b).

PQ caused serum levels of TGF-β1 to increase on day 5 and peak on day 7 after oral ingestion. However, no significant changes of serum TGF-β1 levels were observed in rats treated with thalidomide after PQ exposure (Fig. 2c).

Serum COL1A1 levels in rats of PQ group significantly elevated on day 3, peaked on day 7 and remained higher than control on day 15. Thalidomide treatment completely reversed the PQ-induced increase of COL1A1 levels (Fig. 2d).

### Effects of thalidomide on mRNA expression levels of IL-6, TNF-α, TGF-β1 and COL1A1 in lung tissues after PQ intoxication

**Fig. 3:**
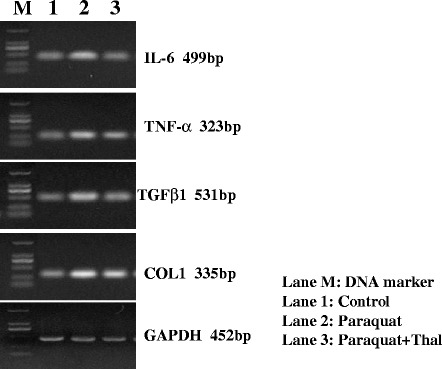
Effects of thalidomide on expression levels of IL-6, TNF-α, TGF-β1 and COL1A1 in lung tissues after PQ intoxication. Lung tissues from each treatment group were excised on day 15 after PQ administration and mRNA expression levels of IL-6, TNF-α, TGF-β1 and COL1A1 genes were examined using RT-PCR

Expression levels of IL-6, TNF-α, TGF-β1 and COL1A1 mRNA transcripts in lung tissues were examined using RT-PCR on day 15 after PQ administration (Fig. 3). Expressions of IL-6 and TNF-α mRNA were significantly up-regulated in lung tissues in PQ group compared to control, indicating the involvement of inflammatory cytokines in PQ-induced pathological changes of lung. In addition, expressions of TGF-β1 and COL1A1, two important fibrosis factors, were also significantly elevated at mRNA level in lung tissues at 15 days after PQ exposure. However, expressions of IL-6, TNF-α, TGF-β1 and COL1A1 transcripts were all significantly lower in lung tissues from rats treated with thalidomide after PQ exposure compared to PQ group. These results indicate that thalidomide may prevent PQ-induced pulmonary fibrosis through downregulating the expression of TGF-β 1, TNF-α and IL-6 in lung tissues.

### Immunohistochemistry detection of the expression of IL-6, TNF-α, TGF-β1 and COL1A1 proteins in lung tissues after PQ intoxication

**Fig. 4:**
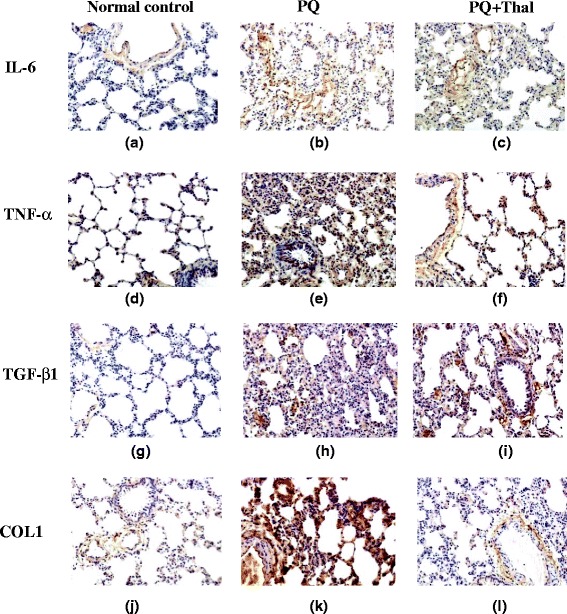
Immunohistochemistry detection of the expression of IL-6, TNF-α, TGF-β1 and COL1A1 proteins in lung tissues after PQ intoxication. Lung tissues from Control (**a**, **d**, **g**, **j**), PQ (**b**, **e**, **h**, **k**) and PQ + Thal (**c**, **f**, **i**, **l**) groups were collected on day 15 after PQ administration and expression of IL-6 (**a**, **b**, **c**), TNF-α (**d**, **e**, **f**), TGF-β1 (**g**, **h**, **i**) and COL1A1 (**j**, **k**, **l**) in lung tissues were examined using immunohistochemical staining (×100)

Expression of IL-6, TNF-α, TGF-β1 and COL1A1 proteins in lung tissues were examined using immunohistochemical staining on day 15 after PQ administration. As shown in Fig. 4, there were weak and diffused signals of IL-6 and TNF-α on vascular walls and alveolar walls in normal lung tissues from control group. Enhanced signals of IL-6 and TNF-α stainings were observed on alveolar walls in lung tissues from PQ group on day 15 post PQ exposure and thalidomide treatment significantly reduced IL-6 and TNF-α expression in lung tissues. Expression of TGF-β1, which was detected at low levels on alveolar walls in normal lung tissues, was increased in lung tissues from PQ group and significantly reduced by thalidomide treatment (Fig. 4). In normal lung tissues, COL1A1 is mainly detected on vascular walls and weakly expressed on alveolar walls. Although expression of COL1A1 in alveolar walls was dramatically increased after PQ exposure, thalidomide treatment resulted in significantly decreased COL1A1 expression in lung tissues compared to PQ group.

## Discussion

Intoxication of paraquat, a highly toxic quaternary ammonium herbicide, has a high mortality rate and poor prognosis due to severe acute lung injury and subsequent pulmonary fibrosis [15]. In this study we have demonstrated that thalidomide has a protective effect against PQ-induced lung injury and pulmonary fibrosis in rats. Our results showed that administration of thalidomide at 60 mg/kg could significantly reduce the degree of lung edema and pulmonary fibrosis caused by PQ in rats. Moreover, our data also suggested that the protective effect of thalidomide against PQ-induced pulmonary fibrosis was associated with decreased expression levels of inflammatory factors such as TGF-β1, TNF-α and IL-6 in lung tissues after PQ exposure.

Lung is one of the major organs affected by PQ during PQ intoxication due to the accumulation of PQ in the lung through the active polyamine uptake system in the membrane of alveolar cell [1, 3]. Although the molecular mechanism of PQ-induced lung injuries is not fully understood, oxidative and inflammatory mediators have been found to play important roles in this process. It has become increasingly clear that reactive oxygen species generated through the cyclic oxido-reduction of PQ can not only result in tissue damages directly but also initiate and promote inflammatory responses through upregulation of inflammatory cytokines and adhesion molecules [16–18]. Such inflammatory cytokines, particularly IL-6, TNF-α, TGF-β1 are found to be involved in the pathogenesis of PQ-induced pulmonary inflammation and subsequent fibrosis [4, 19, 20]. IL-6 and TNF-α are cytokines that are released in both early acute inflammatory and late fibrotic phases of PQ-induced lung injury [4]. TGF-β1 as the most important fibrogenic growth factor has been shown to stimulate fibroblast proliferation, matrix protein production and collagen synthesis [21]. Increased production of TGF-β1 from lung injury caused by PQ has been associated with inflammation and fibrosis of lung tissue [19, 20]. In this study, we observed that the serum levels of IL-6 and TNF-α quickly elevated on day 1, peaked on day 3 and remained higher than control on day 5 after PQ exposure in rats. However, serum TGF-β1 level was not increased until day 5 and peaked on day 7 after PQ exposure. In addition, immunohistochemistry detection for the expression of IL-6, TNF-α and TGF-β1 in lung tissues from rats on day 15 after PQ intoxication showed significant overexpression of these factors in lung tissues compared to control normal lung tissues. Our results, consistent with other studies [4, 19, 20], demonstrate that inflammatory factors play important roles in PQ-induced lung injuries with proinflammatory factors IL-6 and TNF-α involved in both acute and chronic phases and TGF-β1 mainly in late fibrotic phase of PQ-induced lung injury.

Thalidomide and its analogs have been used in clinic as immunomodulatory drugs with anti-inflammatory, anti-proliferative and anti-angiogenic activities. It has been shown that thalidomide exhibits anti-inflammatory and anti-fibrotic activities by suppressing the production of proinflammatory cytokines and growth factors that play important roles in the tissue destruction and fibrosis in chronic inflammatory situations [19, 22, 23]. Several studies using animal models have demonstrated that thalidomide has therapeutic effects for fibrotic and inflammatory disorders such as bleomycin-induced lung fibrosis [14], peritonitis [23], pancreatitis [24], experimental diabetes [22] and liver cirrhosis [25]. Moreover, thalidomide was recently shown to protect against PQ-induced lung injury through different mechanisms in PQ-intoxication mouse models [26–28]. Amirshahrokhi [26] showed that 6 days treatment of thalidomide in PQ intoxicated mice resulted in decreased production of inflammatory and fibrogenic cytokines including TNF-α, IL-1β, IL-6, and TGF-β1 as well as reduced myeloperoxidase (MPO), nitric oxide (NO), and hydroxyproline content in lung tissues. In another two published studies performed by a Chinese group [27, 28], thalidomide was shown to attenuate PQ-induced acute lung injury through multiple mechanisms which involved downregulation of TNF-α, IL-1β, and IL-6 via inhibition of NF-κB activation as well as protection from PQ-induced lipid peroxide damage by activation of the Nrf2-ARE signaling pathway. In the current study we employed a 15-day thalidomide treatment regimen in the PQ intoxication rat model to evaluate the effects and potential mechanisms of thalidomide on PQ-induced lung injury, especially pulmonary fibrosis subsequent to acute lung injury phase. Our results showed that thalidomide treatment after PQ administration resulted in improved survival and rats treated with thalidomide had significantly reduced lung edema and improved pulmonary function at 15 days after treatment. Histological analysis showed that PQ-induced lung injuries characterized by a marked thickening of the alveolar septum and infiltration of various inflammatory cells were attenuated by administration of thalidomide. Lung fibrosis and collagen deposition caused by PQ was significantly relieved after 15-day thalidomide treatment as indicated by Masson’s Trichrome staining. To explore the mechanisms of the protective effect of thalidomide on PQ-induced lung injury, we examined the expression levels of several inflammatory factors including TNF-α, IL-6 and TGF-β1 that play important roles in pathogenesis of PQ-induced lung damages in both blood and lung tissues. Our results showed that blood levels of TNF-α and IL-6 elevated to lower levels and reduced to normal in a shorter time after PQ exposure in rats treated with thalidomide as compared to control PQ intoxicated rats without thalidomide treatment. TGF-β1 as one of the major fibrogenic factors in PQ-induced pulmonary fibrosis was significantly upregulated in blood at day 5 after PQ exposure, while thalidomide treatment completely reversed PQ-induced increase of TGF-β1 level in blood. Similarly, increase of blood COL1A1 level due to PQ exposure was also corrected by thalidomide treatment. These results indicated that immediate treatment with thalidomide after PQ intoxication could interfere with the onset of inflammatory and fibrogenic process and therefore significantly ameliorate pulmonary fibrosis. In addition, immunohistochemistry staining and PCR detection for expression of TNF-α, IL-6 and TGF-β1 in lung tissues on day 15 after PQ intoxication further confirmed that thalidomide could significantly reduce the upregulation of the expression of these factors induced by PQ at both protein and RNA levels. Our findings together with others [26–28] indicate that thalidomide may exhibit anti-inflammatory and anti-fibrosis effects in a non-specific way by regulating the expression of several key inflammatory factors, such as TNF-α, IL-6 and TGF-β1. However, more sophisticated studies are needed to further identify potential signaling pathways and molecular mechanisms of thalidomide’s anti-inflammatory and anti-fibrosis effects against PQ-induced lung injury.

## Conclusions

Thalidomide exhibits protective effect against PQ-induced acute lung injury and subsequent fibrosis by inhibiting the upregulation of inflammatory factors, particularly IL-6, TNF-α and TGF-β1 in lung. Since PQ-induced lung damage begins at a very early stage of PQ poisoning, the treatment with thalidomide should be applied as early as possible after PQ exposure. Our results support clinic trials to explore whether adding thalidomide to current treatment regimen can improve the outcomes of patients with PQ intoxication.

## Abbreviations

PQ: Paraquat
Thal: Thalidomide
TNF-α: Tumor necrosis factor-alpha
IL-6: Interleukine-6
IL-1β: Interleukine-1 beta
TGF-β1: Transforming growth factor-beta 1
H&E: Hematoxylin-and-eosin
COL1A1: Collagen type I, alpha 1
